# iTRAQ-Based Quantitative Proteomic Analysis of the Arabidopsis Mutant *opr3-1* in Response to Exogenous MeJA

**DOI:** 10.3390/ijms21020571

**Published:** 2020-01-16

**Authors:** Jiayu Qi, Xiaoyun Zhao, Zhen Li

**Affiliations:** State Key Laboratory of Plant Physiology and Biochemistry, College of Biological Sciences, China Agricultural University, Beijing 100193, China; qijiayu97@163.com (J.Q.); xiaoyunzhao@cau.edu.cn (X.Z.)

**Keywords:** jasmonic acid, *opr3*, stress defense, quantitative proteomics

## Abstract

Jasmonates (JAs) regulate the defense of biotic and abiotic stresses, growth, development, and many other important biological processes in plants. The comprehensive proteomic profiling of plants under JAs treatment provides insights into the regulation mechanism of JAs. Isobaric tags for relative and absolute quantification (iTRAQ)-based quantitative proteomic analysis was performed on the Arabidopsis wild type (Ws) and JA synthesis deficiency mutant *opr3-1*. The effects of exogenous MeJA treatment on the proteome of *opr3-1*, which lacks endogenous JAs, were investigated. A total of 3683 proteins were identified and 126 proteins were differentially regulated between different genotypes and treatment groups. The functional classification of these differentially regulated proteins showed that they were involved in metabolic processes, responses to abiotic stress or biotic stress, the defense against pathogens and wounds, photosynthesis, protein synthesis, and developmental processes. Exogenous MeJA treatment induced the up-regulation of a large number of defense-related proteins and photosynthesis-related proteins, it also induced the down-regulation of many ribosomal proteins in *opr3-1*. These results were further verified by a quantitative real-time PCR (qRT-PCR) analysis of 15 selected genes. Our research provides the basis for further understanding the molecular mechanism of JAs’ regulation of plant defense, photosynthesis, protein synthesis, and development.

## 1. Introduction

Jasmonic acid (JA) is a plant hormone that plays an integral role in the regulation of plant growth and development as well as in plant defense against wounding, herbivory attack, and other biotic and abiotic stresses [1]. Jasmonates (JAs) are a class of compounds derived from jasmonic acid with varying biological activities, including the active form (3R,7S) jasmonoyl-isoleucine (JA-Ile) and the volatile methyl ester form methyl jasmonate (MeJA) [2,3]. As a stress-related hormone, JAs are involved in plant defense against insects and pathogens [4,5], the response to ultraviolet radiation [6], drought, and other abiotic stresses [7,8,9,10]. In unwounded plant tissues, JAs regulate plant growth and development [11], affect root growth [12], senescence, and stamen development [13,14,15,16,17]. However, when encountering insect herbivory attack, plants immediately undergo a series of physiological responses and initiate a rapid biosynthesis of JAs to trigger the transduction of a stress signal, which results in the activation of the wound defense mechanism [18,19,20]. Most plants derive their jasmonates from octadecanoid (18-carbon) fatty acid, while some plants also produce jasmonates from hexadecanoid (16-carbon) fatty acid [21]. The major JA synthesis pathway starts from α-linolenic acid (18:3) released from membrane lipids. The linolenic acid is oxygenated by 13-lipoxygenase (LOX) to form 13-hydroperoxylinolenic acid (13-HPOT) in the plasma membrane. The resulting fatty acid hydroperoxide is released from the plasma membrane to peroxisome, which is then dehydrated by allene oxide synthase (AOS) and cyclized by allene oxide cyclase (AOC) to form the cyclopentenone 12-oxo-phytodienoic acid (9S,13S-OPDA). The pentacyclic ring double bond in 9S,13S-OPDA is reduced by OPDA reductase 3 (OPR3) in the peroxisome to form 8-(3-oxo-2 (pent-2-enyl)-cyclopentyl) octanoic acid (OPC:8). Finally, 3R,7S-JA is generated from OPC:8 after three cycles of β-oxidation in the cytoplasm [22,23]. JA can also be produced from the OPDA derivative 4,5-didehydro-JA when OPR3 is completely knocked-out [24]. JA can be catalyzed by JA carboxyl methyltransferase (JMT) to form MeJA, while methyl jasmonate esterase (MJE) can convert MeJA to form JA. The active form of jasmonic acid, JA-Ile, is formed through the conjugation of jasmonic acid with isoleucine and is perceived by the COI1/JAZ co-receptor [25,26]. MeJA is volatile and can penetrate through plasma membrane readily, and it can be quickly converted to JA and eventually to JA-Ile to participate in systemic signaling during development and responses to stress [27]. MeJA has been widely used to study jasmonates signaling pathways and the mechanisms of plant defense.

Mutants defective in JA biosynthesis and response have been used to investigate the roles of JAs in defense and development [28,29,30,31]. These mutants included *dad1, fad3-3, fad7-2, fad8, dde2-2, dde1*, and *opr3*. OPR3 is one of the restrictive enzymes in the JA synthesis pathway. There are six OPR enzymes in Arabidopsis, but only OPR3 can effectively catalyze the reduction of 9S,13S-OPDA in plants [32,33,34,35]. Endogenous JA is nearly absent in *opr3-1* and the mutant showed three characteristic phenotypes: floral organs develop normally within the closed bud, but the anther filaments do not elongate enough to reach the locules above the stigma at the anthesis stage; the anther locules do not dehisce during flowering; the pollen grains are predominantly inviable [36]. However, *opr3-1* is not a null mutant, it can form mature full-length *OPR3* transcripts and synthesize JA under specific conditions like under B. cinerea infection [37]. In the OPR3 complete knock-out mutant (*opr3-3*), JA can still be synthesized through OPDA derivative 4,5-didehydro-JA under the catalysis of OPR2 [24]. Exogenous MeJA treatment can restore stamen development, the inhibition of root growth and the degradation of the jasmonate repressor JAZ1 in JA biosynthesis-deficient mutants [24], such as *opr3*, but not in JA signaling-deficient mutants [24]. Compared with other JA synthesis defect mutants, the *opr3-1* mutant is more resistant to necrotrophic fungus, Alternaria brassicicola, as well as to the soil gnat, Bradysia impatiensthus [37]. So, *opr3-1* is a valuable model to investigate the mechanism of the JA signaling pathway due to its nearly absent endogenous JAs [36,38]. It has been reported that exogenous JAs were involved in fertility regulation and root growth [39,40], whether the application of exogenous JAs on biosynthesis-deficient mutants, such as *opr3-1*, affects other functions of Arabidopsis remains an intriguing research topic.

The fast development of transcriptics and mass spectrometry-based quantitative proteomics approaches provide powerful tools to investigate the biological response of plants under external stress conditions or exogenous hormone stimulus. Pauwels L et al. investigated alterations in the transcriptome of the fast-dividing cell culture of Arabidopsis after exogenous MeJA treatment. The results showed early MeJA response genes encoded the JA biosynthesis pathway proteins and key regulators of MeJA responses, including most JA ZIM domain proteins and MYC2, meanwhile, in the second transcriptional wave, MeJA response transcripts were mainly involved in cellular metabolism and cell cycle progression [41]. Mata-Perez et al. used RNA-seq to study the profiles of Arabidopsis cell suspension cultures transcriptomes after linolenic acid treatment, identified 533 up- and 2501 down-regulated genes. RNA-seq data analysis showed that an important set of these genes was associated with the JA biosynthetic pathway, including LOX and AOC. In addition, several transcription factor families involved in the response to biotic stresses, such as pathogen attacks or herbivore feeding, were identified [42]. Guo et al. used an iTRAQ-based quantitative proteomics approach to analyze broccoli sprouts treated with exogenous jasmonic acid and found that photosynthesis and protein synthesis were inhibited after JA treatment, which was responsible for the slower growth of broccoli, but carbon metabolism and amino acid metabolism-related proteins were up-regulated [43]. Farooq et al. investigated MeJA-induced Arsenic tolerance in *Brassica napus* leaves using iTRAQ and 110 differentially regulated proteins were identified—proteins that were involved in stress and defense, photosynthesis, carbohydrates and energy production, protein metabolism, and secondary metabolites [44]. Alvarez et al. investigated the changes in protein redox regulation in response to oxidative stress induced by MeJA in Arabidopsis shoots and roots using quantitative proteomics approach and confirmed cysteine residues of proteins were involved in redox regulation, which provided a deeper understanding of the jasmonate signaling and regulation network [45].

Most of the reports investigated the effects of exogenous JAs on stress and defense responses in the presence of endogenous JA. There were very limited reports on the effects of exogenous JAs in the absence of endogenous JAs. The recovery of fertility in *opr3-1* after exogenous MeJA treatment indicated that exogenous JAs can, at least partially, replace the role of endogenous JA. Thus, we raise the following question: which signaling pathways and metabolic processes can be affected by exogenous JAs in the absence of endogenous JAs? 

In this study, we used an iTRAQ-based quantitative proteomic method to investigate the effects of exogenous MeJA on JA synthesis deficient mutant *opr3-1* (Figure S1). A total of 126 differentially regulated proteins (DRPs) were identified between the control and treatment groups of both genotypes (Arabidopsis wild type (Ws) and *opr3-1*) after MeJA treatment. These DRPs were involved in metabolism processes, responses to stress, the defense against pathogens and wounds, photosynthesis, protein synthesis, as well as development processes. The transcriptional level of 15 selected genes from the DRPs was further validated by qRT-PCR analysis. Our work contributed to a better understanding of the molecular mechanisms of JAs regulating plant defense, photosynthesis, protein synthesis, and development.

## 2. Results

### 2.1. Overview of Protein Identified in Ws and opr3-1

**Figure 1:**
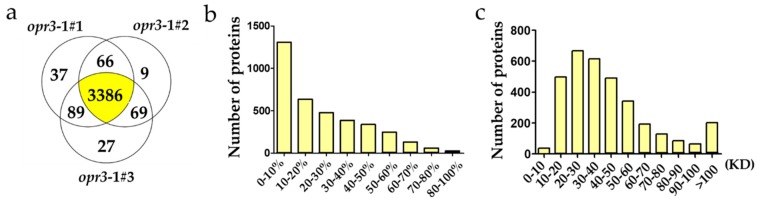
Information on the identified proteins. (**a**) A Venn diagram of the number of proteins identified in three replicates; (**b**) the distribution of sequence coverage; (**c**) the distribution of the mass of the identified proteins.

A quantitative proteomics analysis of Arabidopsis wild type (Ws) and JA synthesis deficient mutant (*opr3-1*) treated with 0.25 mM of MeJA for 8 h was performed to identify differentially expressed proteins between these genotypes under exogenous MeJA treatment. A total of 45,691 unique spectra corresponding to 25,957 unique peptides and 3683 proteins were identified in this experiment. Among them, 3386 proteins can be identified in all three replicates and 3214 proteins can be quantified (Figure 1a). It can be seen that sequence coverages of the identified proteins were mostly below 30%, and most of the identified proteins were in the mass range 20–30 KD and 30–40 KD (Figure 1b,c).

### 2.2. Identification of Differentially Regulated Proteins

**Figure 2:**
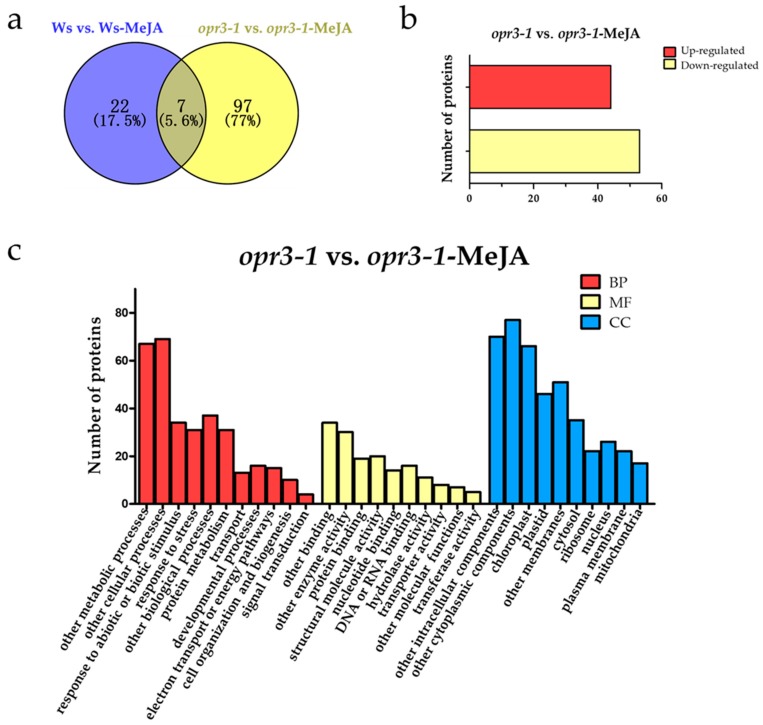
Functional classifications of differentially regulated proteins. (**a**) A Venn diagram of differentially regulated proteins; (**b**) the numbers of up-regulated and down-regulated proteins; (**c**) the GO assignment of DRPs in *opr3-1* in response to methyl jasmonate (MeJA) treatment (*opr3-1*-MeJA). BP: Biological Process; MF: Molecular Function; CC: Cellular Component.

In order to explore the effect of exogenous JAs on *opr3-1* at the proteome level, differentially regulated proteins (DRPs, fold change > 1.5, *p* < 0.05) were screened according to the intensity of the iTRAQ reporter ions. A total of 126 DRPs were identified between the control and treatment groups of both genotypes (Figure 2a). To further understand the effects of exogenous JAs on the proteome of Arabidopsis in the absence of endogenous JA, we screened DRPs between *opr3-1* and *opr3-1* after the MeJA treatment and removed the proteins that showed significant changes in abundance in Ws after MeJA treatment. The remaining 97 DRPs were considered as proteins that were induced by exogenous JAs. Among them, 44 proteins were up-regulated and 53 proteins were down-regulated (Figure 2b). These DRPs were used for the following functional analysis.

### 2.3. Functional Analysis of Differentially Regulated Proteins

GO analysis of the DRPs showed that the DRPs responded to MeJA in *opr3-1* can be classified into 11 biological process categories: metabolic processes (20.48%), cellular processes (21.10%), the response to abiotic or biotic stimulus (10.39%), the response to stress (9.48%), other biological processes (11.31%), protein metabolism (9.48%), transport (3.97%), developmental processes (4.89%), electron transport or energy pathways (4.58%), cell organization and biogenesis (3.05%), and signal transduction (1.22%). For molecular functions, 20.73% of the proteins were related to binding activity, followed by enzyme activity (18.29%), structural molecule activity (12.19%), protein binding (11.58%), DNA or RNA binding (9.76%), nucleotide binding (8.54%), hydrolase activity (6.71%), transporter activity (4.88%), other molecular functions (4.27%), and transferase activity (3.05%). In the cellular components category, 17.82% of the DRPs were cytoplasmic components, followed by intracellular components (16.20%), chloroplast (15.28%), other membranes (11.81%), plastids (10.65%), cytosol (8.10%), nucleus (6.02%), ribosome (5.09%), plasma membrane (5.09%), and mitochondria (3.93%) (Figure 2c). 

A Kyoto Encyclopedia of Genes and Genomes (KEGG) pathway analysis of the DRPs between *opr3-1* and *opr3-1*-MeJA showed 20 functional classes (Figure S2). Most of the proteins were enriched in metabolic pathways, protein synthesis, photosynthesis, the biosynthesis of secondary metabolites, carbon metabolism, and the biosynthesis of amino acids.

**Figure 3:**
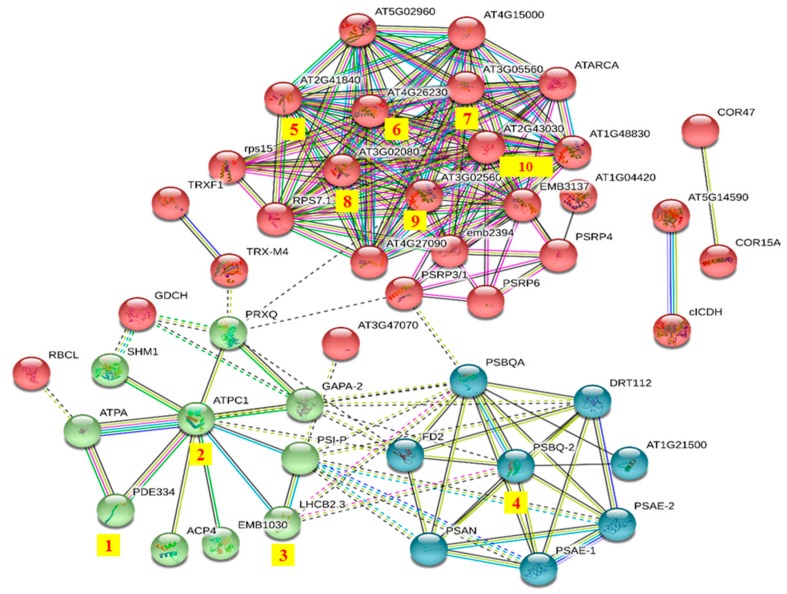
Protein–protein interaction network of differentially regulated proteins (DRPs) in *opr3-1* under MeJA-treatment. Red group: protein synthesis; green group: energy metabolism; blue group: photosynthesis.

A STRING analysis was performed to investigate the interaction network among these DRPs. The DRPs can be divided into three groups (Figure 3). They were involved in protein synthesis (red group), energy metabolism (green group), and photosynthesis (blue group). Among these proteins, ATP synthase gamma chain 1 (AT4G04640.1, No.2) is involved in the regulation of ATPase activity, it catalyzes the conversion of ATP from ADP in the presence of a proton gradient across the membrane [46]. The abundance of this protein decreased by 0.59 fold in *opr3-1* after MeJA treatment, indicating that MeJA treatment reduced the synthesis of ATP and impaired the energy metabolism of *opr3-1*. An oxygen-evolving enhancer protein (AT4G05180.1, No.4) is required for photosystem II assembly/stability. The loss of the oxygen-evolving enhancer protein induces significant decreases in photosystem II function [47], and this protein was up-regulated by 1.80 folds after MeJA treatment in *opr3-1*. The expression of the Chlorophyll a–b binding protein (AT3G27690.1, No.3) was increased by 1.54 folds in *opr3-1* after MeJA treatment. This protein acts as a light receptor and is closely related to photosystems. The up-regulation of these two proteins in *opr3-1* after MeJA treatment indicated that MeJA treatment could enhance photosynthesis in *opr3-1*. When the plant is mechanically damaged, the JAs’ content increases abruptly [48], while the application of exogenous MeJA simulates the process of pest or bacteria invasion and leaf damage, which results in the activation of the JAs’ synthesis pathway, however, in the *opr3-1* mutant, the in vivo synthesis of JA is inhibited due to the lack of OPR3 enzyme, thus, the *opr3-1* mutant provides an excellent model to investigate the effects of exogenous JAs without background interference from endogenous JAs. We found that the abundance of pigment defective 334 (AT4G32260.1, No.1), which has hydrogen ion transmembrane transporter activity and is involved in the defense response to bacterium, was increased by 1.66 folds in *opr3-1* after MeJA treatment. The up-regulation of this protein suggested that the application of exogenous MeJA could induce the defense mechanism against bacterium invasion. Proteins in the red group (protein synthesis-related process) were closely interconnected, these proteins (AT2G41840.1, AT4G26230.1, AT3G05560.1, AT3G02080.1, AT3G02560.2, AT2G43030.1, No.5—10) mostly belonged to the ribosomal protein family and were involved in translation. The abundance of these proteins decreased by 0.66, 0.65, 0.61, 0.64, 0.66, and 0.65 folds in *opr3-1* after MeJA treatment, respectively. The down-regulation of these proteins indicated that MeJA treatment inhibited protein synthesis in *opr3-1*. 

### 2.4. Verification of the DRPs by qRT-PCR

**Figure 4:**
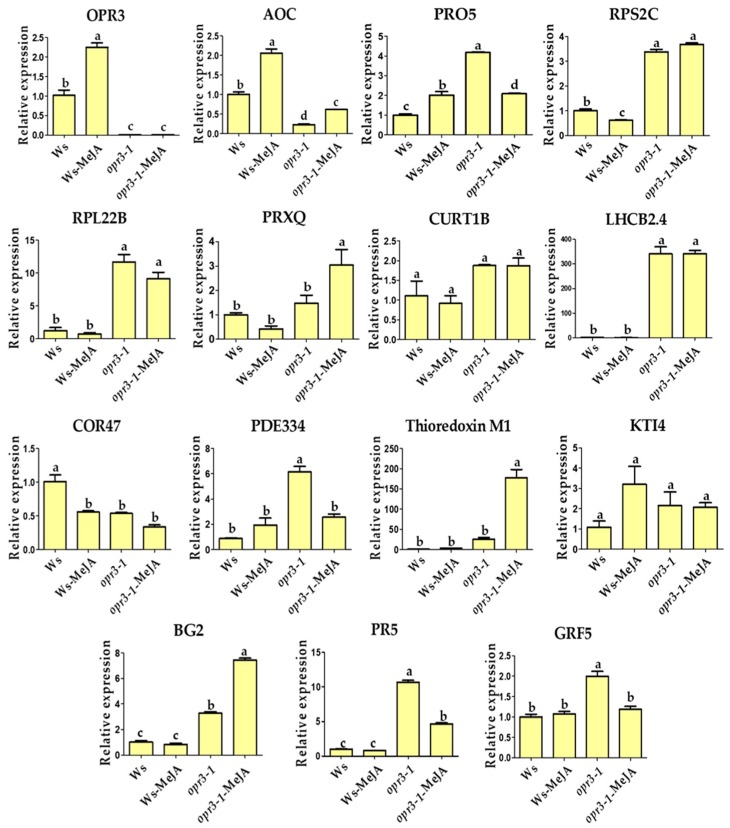
Relative mRNA expression levels of selected genes measured by qRT-PCR. OPR3: 12-oxophytodienoate reductase 3; AOC: Allene oxide cyclase 1; PRO5: Profilin-5; RPS2C: 40S ribosomal protein S2-3; RPL22B: 60S ribosomal protein L22-2; PRXQ: Peroxiredoxin Q; CURT1B: Curvature thylakoid 1B; LHCB2.4: Chlorophyll a-b binding protein 2.4; COR47: Dehydrin COR47; PDE334: Pigment defective 334; Thioredoxin M1: Arabidopsis thioredoxin m-type 1; KTI4: Kunitz trypsin inhibitor 4; BG2: Glucan endo-1,3-beta-glucosidase; PR5: Pathogenesis-related protein 5; GRF5: 14-3-3-like protein GF14 upsilon.

To validate the iTRAQ results, the transcriptional levels of 15 candidate DRPs were analyzed using qRT-PCR (Figure 4). Among them, six DRPs showed similar trends of variation in their mRNA expression level compared with protein expression, including proteins involved in JA synthesis (OPR3, AOC), photosynthesis (PRXQ), protein domain specific binding (GRF5), and defense against pathogens and wounds (BG2, Thioredoxin M1) (Figure 4). *OPR3* and *AOC* are key genes in the synthesis of the JA pathway, *OPR3* was not expressed in the *opr3-1* mutant as expected, the expression of *AOC* was significantly increased after MeJA treatment in both genotypes, but the expression level was generally lower in the *opr3-1* mutant compared with the wild type (Figure 4). However, the alteration in protein expression levels did not always correlate well with the changes in mRNA expression. In this study, we found several genes with discrepancies in protein and mRNA abundances. For example, the abundance of RPS2C and RPL22B (protein synthesis-related) decreased in *opr3-1* after MeJA treatment, while their mRNA expression showed no significant changes in *opr3-1*. MeJA treatment resulted in accumulations of PDE334 and PR5 (related to defense against pathogen) in *opr3-1* but their mRNA expression decreased in *opr3-1* after treatment. Such discrepancies between qRT-PCR and iTRAQ results can be attributed to the post-transcriptional, translational, and post-translational regulation of gene expression [49]. 

## 3. Discussion

**Figure 5:**
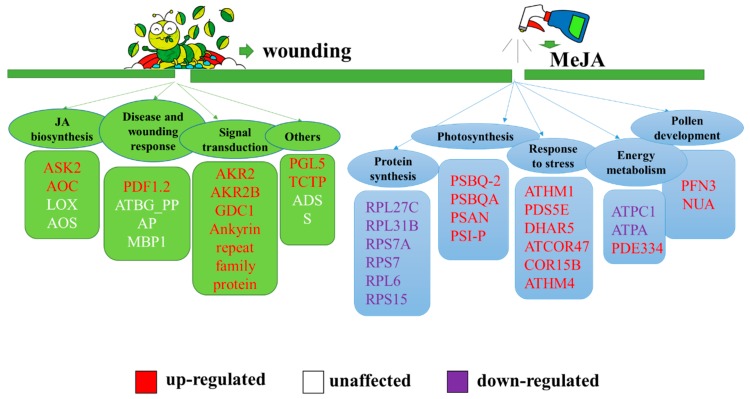
Summary of the biological processes affected by exogenous MeJA in the absence of endogenous jasmonic acid (JA). The red: up-regulation of protein expression; white: no significant change; purple: down-regulation of protein expression. Green group: proteins affected by JAs reported previously; blue group: proteins affected by jasmonates (JAs) discovered in this study. Orange group: plant hormone affected by JAs in this study.

Jasmonates, including jasmonic acid, methyl jasmonate, and jasmonoyl-isoleucine are crucial plant hormones widely present in higher plants. They play important roles in regulating seed germination, growth, pollen fertility, the response to external damage (mechanical, herbivore, insect damage) and pathogenic infections. The endogenous level of JA in *opr3-1* was only about 1/5 of the wild type (Figure S5). The deficiency of endogenous JAs in the *opr3-1* mutant results in the anther filaments not elongating enough to reach the stigma, anther locules not dehiscing, and inviable pollen grains, which eventually results in male sterility. The application of exogenous JAs can restore the male sterile phenotype. The nearly absence of endogenous JAs in *opr3-1* provide an excellent model to investigate the regulation mechanism of JA on plant development and defense response. Previous studies mostly focused on changes in the gene expression levels induced by exogenous JA, while changes at the proteomic levels were less explored [50,51]. Therefore, we used an iTRAQ-based quantitative proteomics approach to identify responsive proteins in JA synthesis deficient mutants, after exogenous MeJA treatment, as a means of exploring the regulatory roles of JAs. This study not only discovered the classic JA-induced proteins reported in previous studies [52,53] but also discovered some new proteins affected by exogenous MeJA, which are mainly involved in protein synthesis, photosynthesis, the response to stress, energy metabolism, and pollen development (Figure 5). 

### 3.1. MeJA-Induced Physiological Changes

The physiological assays showed that there was no significant difference in the content of H_2_O_2_ between Ws and *opr3-1* under normal condition (Figure S3a,b). After 8 h of MeJA treatment, the H_2_O_2_ content in Ws increased, while the opposite trend was observed in *opr3-1* (Figure S3c). In addition, *opr3-1* had higher Peroxidase (POD) content under normal conditions, and the content of POD in *opr3-1* was almost two times higher than that of Ws. The MeJA treatment led to decreases in POD content in both genotypes and a larger decrease in *opr3-1* was observed (Figure S3d), but *opr3-1* still managed to maintain a higher POD content than Ws. These results indicate that *opr3-1* can maintain a better reactive oxygen species (ROS) scavenging capability under exogenous MeJA treatment. The higher POD content in *opr3-1* may contribute to its lower ROS level, even though the POD content decreased in both genotypes after MeJA treatment. In the proteomics data, it was found that the abundances of ROS scavenge-related proteins (AT1G19570.1, AT3G26060.1, AT1G03680.1) were up-regulated in *opr3-1* after MeJA treatment, which may also explain the decrease in ROS content in *opr3-1*. 

The contents of the free amino acid of the two genotypes were determined by liquid chromatograph-mass spectrometer (LC-MS) after derivatization with the AccQ tag reagent and the effect of exogenous MeJA treatment on free amino acids was revealed by principal component analysis (Figure S4). There were significant differences in free amino acid contents between Ws and *opr3-1* under normal conditions, and MeJA treatment did not show a significant effect on amino acids’ contents in both genotypes. These data show that there was a significant difference in endogenous free amino acids’ contents between Ws and *opr3-1*. Such a discrepancy cannot be compensated by the exogenous application of MeJA, indicating that exogenous JAs cannot fully replace the functions of endogenous JAs.

The contents of three major plant hormones (ABA, JA, SA) were measured using LC-MS in Ws and *opr3-1* before and after MeJA treatment (Figure S5). SA, JA, and ABA play important roles in plant defense and stress response. SA is best known for its central role in the plant defense response against pathogens and as an inducer of systemic acquired resistance. It is synthesized from chorismate via isochorismate. The infection of plants by pathogens results in an increase in SA levels both at the site of infection and in distant tissues [54]. ABA is an isoprenoid compound associated with seed dormancy, drought responses, and other growth processes [55]. ABA levels are regulated by a variety of environmental factors, including drought, cold, and other biotic or abiotic stresses [56]. In response to abiotic stress, the crosstalk between plant hormonal signaling pathways prioritizes defense over other cellular functions [57]. SA and JA-mediated signaling pathways are closely related in plant stress resistance, and they crosstalk through certain regulatory factors, such as *NPR1* [58]. The antagonism between SA and JA signaling pathways results in the downregulation of a large set of JA-responsive genes, including the marker genes PDF1.2 and VSP2 in the presence of SA [59]. In this study, the significantly reduced JA content in *opr3-1* confirmed the mutation of the *OPR3* gene, and the spike in JA content after exogenous MeJA treatment indicated a quick absorption and transformation of MeJA (Figure S5). Moreover, the ABA and SA contents showed similar trends after MeJA treatment, i.e., their contents were both significantly higher in *opr3-1* than in Ws under normal conditions, and they were both significantly decreased after MeJA treatment. Also, the plant hormone contents in MeJA-treated *opr3-1* was similar to those of the untreated wild type. These data indicate that there was antagonism between JA and ABA/SA in both Ws and *opr3-1*, and the lack of endogenous JA resulted in higher ABA and SA levels in untreated *opr3-1*, while the application of MeJA reduced the levels of these two hormones. 

### 3.2. Proteins Response to MeJA Treatment in opr3-1

#### 3.2.1. Stress-Related Proteins

When wounded or under insect or pathogen attack, plants initiate defensive mechanisms and activate the JA synthesis pathway, which results in a sharp increase in JA content [60]. In this experiment, the application of exogenous MeJA simulated such stress process in the plants. We found four wound-related proteins, DHAR1 (AT1G19570.1), ATCOR47 (AT1G20440.1), PDE334 (AT4G32260.1), and ATHM1 (AT1G03680.1). The abundance of these proteins were up-regulated by 1.62, 1.70, 1.66, and 1.51 folds, respectively, in *opr3-1*, after MeJA treatment. DHAR1 is a key component of the ascorbate recycling system; it is involved in ROS scavenging under oxidative stresses [61]. ATHM1 is the key enzyme of the oxidative pentose phosphate pathway, which supplies reducing power (as NADPH) in non-photosynthesizing cells, and is involved in the response to oxidative stress and regulates the carbohydrate metabolic process [62]. The up-regulated expression of DHAR1 and ATHM1 indicates the enhanced ROS scavenging capability of *opr3-1* in the presence of exogenous MeJA, which is evidenced by reduced H_2_O_2_ level in MeJA-treated *opr3-1* (Figure S3c). The accumulation of ATCOR47 is triggered in response to the presence of fungus and PDE334 and is involved in the response to an invasion of bacterium [63,64]. The accumulation of these two proteins indicates that the application of exogenous MeJA could also induce the expression of proteins involved in the response to fungus and bacterium invasion.

#### 3.2.2. Pollen Development-Related Proteins

In *opr3-1*, the pollen grains are inviable, the anthers are abnormally dehydrated, and the anther filaments do not elongate, resulting in male sterility. These defects can be remedied by the application of exogenous MeJA, indicating that JA is required for male gamete development [65]. In the proteomic results, we found several up-regulated proteins in response to MeJA in *opr3-1* that were involved in anther and pollen development.

Nuclear pore anchor (NUA, AT1G79280.2) is a component of the nuclear pore complex, it mediates the transportation of RNA and other cargoes between the nucleus and the cytoplasm. Nuclear pore anchor mutants *nua-1* and *nua-4* showed diverse developmental phenotypes, including early flowering, stunted growth, and shortened anther filament [66], indicating that NUA is required for filament elongation. Profilin-3 (AT5G56600.1) is a ubiquitous eukaryotic protein that regulates the actin cytoskeleton, which is essential for pollen development. Profilin-3 can rearrange the actin cytoskeleton during pollen germination, and recently, it has been identified as a potent regulatory factor in pollen development [67]. The up-regulation of these two proteins (1.66 and 1.32 folds) in *opr3-1*, after MeJA treatment, indicates that these two proteins are induced by exogenous MeJA and that they may be curial components for restoring *opr3-1*’s fertility.

#### 3.2.3. Protein Synthesis-Related Proteins

Ribosomes contain a large number of ribosomal proteins, which can catalyze the peptidyl transfer reaction for polypeptide synthesis. They are responsible for protein synthesis and play a major role in regulating cell growth, differentiation, and development [68]. In this study, we found five 40S ribosomal proteins (AT2G41840.1, AT3G02080.1, AT1G48830.1, AT3G02560.1, AT5G02960.1), four 60S ribosomal proteins (AT4G15000.1, AT4G26230.1, AT3G05560.1, AT4G27090.1), two 30S ribosomal proteins (ATCG00900.1, AT5G14320.1), and one 50S ribosomal protein (AT2G43030.1) that were dramatically down-regulated by MeJA in *opr3-1* (Table S1). The decreased abundance of these ribosomal proteins suggests that MeJA treatment inhibited protein synthesis in *opr3-1*.

#### 3.2.4. Photosynthesis-Related Proteins

In plants, photosynthesis is an important metabolic process and is susceptible to environmental stress. It has been reported that photosynthesis rate was promoted in Arabidopsis under drought stress [69]. In this study, iTRAQ data show that MeJA application could remarkably enhance the expression of photosynthesis-related proteins. The photosystem I reaction center subunit N (AT5G64040.2) may function in mediating the binding of the antenna complexes to the PSI reaction center and core antenna. It plays an important role in docking plastocyanin to the PSI complex [47]. Photosystem I protein P (AT2G46820.1) is a part of the photosystem I complex [70]. Photosystem II subunit Q-1 (AT4G05180.1) and photosystem II subunit Q-2 (AT4G21280.2) encode the PsbQ subunit of the oxygen evolving complex of photosystem II. They are required for photosystem II assembly/stability [71]. These proteins were up-regulated by 1.51, 1.95, 1.70, and 1.55 folds in *opr3-1* after MeJA treatment, respectively. The up-regulation of these photosynthesis-related proteins suggests that exogenous MeJA treatment enhances plant photosynthesis processes.

## 4. Materials and Methods

### 4.1. Plant Materials and Growth Conditions

The seeds of *Arabidopsis thaliana* ecotype Wassileskija (Ws) and mutant *opr3-1* were sterilized with 1% NaClO for 10 min, followed by washing with distilled water and sowing onto a 1/2 MS medium for 10 days. Afterwards the seedlings were transferred to pots to a climate chamber (22 °C; 8/16 h light/dark cycle, 65% rh).

Four-week-old Arabidopsis (bolting but not flowering) plants were sprayed with 250 μM MeJA in 0.05% Tween-20, and the control groups were sprayed with 0.05% Tween-20 without MeJA. After 8 h of treatment, the leaves of the plants were collected, ground into a fine powder in liquid nitrogen and stored at −80 °C until protein extraction.

### 4.2. Protein Extraction and Digestion

Arabidopsis leaf proteins were extracted by a modified phenol extraction method [72]. In brief, 0.5 g of leaves were ground into fine powder in liquid nitrogen, and a 3 mL protein extraction buffer (500 mM Tris–HCl, 700 mM sucrose, 500 mM EDTA,100 mM KCl, 1% protease inhibitor cocktail, 1% phospho-STOP, pH 8.0) was added and ground for 10 min. Then, 3 mL of Tris-saturated phenol was added and ground for another 10 min. The phenol layer was collected after centrifugation and the proteins were precipitated with 0.1 M ammonium acetate in methanol overnight at −20 °C. The protein pellet was washed three times with pre-cooled acetone and dried. The protein pellet was dissolved with 7 M urea/2 M thiourea, and the protein concentration was measured by Bradford assay. 

The protein was digested with a modified filter-aided sample preparation (FASP) workflow [73]. In short, 200 μg of protein was loaded onto an ultrafiltration device (10 KDa, MWCO, 500 μL, Sartorius, Gottingen, Germany), reduced with 50 mM DTT at 56 °C and alkylated with 200 mM IAM for 30 min, in the dark, at room temperature. The protein was digested with trypsin with a protein:enzyme ratio of 50:1 at 37 °C for 16 h.

### 4.3. iTRAQ Labeling, High pH Reversed-Phase Fractionation, and NanoLC-MS Analysis

An iTRAQ 8-plex kit was used to label peptides from Ws and *opr3-1*, with or without MeJA treatment, following the manufacturer’s instructions. The details of the iTRAQ channels used for each sample are listed in Table S2. Three biological replicates were analyzed. 

The labeled peptides were pooled and fractionated with a C18 column (2.1 mm × 100 mm, 2.6 μm, Kinetex, Phenomenex) using a gradient elution program of 20 mM ammonium acetate in water (pH 10.0) and 20 mM ammonium acetate in 90% acetonitrile (pH 10.0) on a High Performance Liquid Chromatography (HPLC) system (H-Class bio, Waters, Milford, MA, USA). The peptides were pooled into 12 fractions, dried in a vacuum concentrator and resuspended with 0.1% formic acid.

Protein identification was performed with a Q-Exactive high resolution mass spectrometer (Thermo Fisher Scientific, Waltham, MA, United States) coupled with nanoAcquity HPLC (Waters, Milford, MA, USA). The labeled peptides were loaded on an Acclaim PepMap C18 trap column (75 μm × 2 cm, 3 μm, C18, 100Å, Thermo Fisher Scientific) and separated by a home-made C18 column (100 μm × 15 cm, 3 μm, C18, 125Å, Phenomenex) at a flow rate of 400 nL/min. Peptide elution was achieved through a linear gradient of Buffer B (0.1% formic acid in acetonitrile) in 120 min. An MS survey scan was performed between 300–1800 m/z with a resolution of 70,000. Higher energy collisional dissociation (HCD) fragmentation was performed for the 10 most intensive precursor ions with a resolution of 17,500, and the dynamic exclusion time was 30 s.

The MS raw files were processed with a Mascot distiller and searched with Mascot (version 2.6.0, Matrix Science, London, United Kingdom) against the TAIR10 database. Scaffold Q+ (version 4.8.7, Proteome Software, Portland, OR, United States) was used for quantitative analysis. The search parameters were as follows: enzyme specificity was set as trypsin with two missed cleavages; precursor ion mass tolerance was set at 10 ppm and MS/MS fragment ion mass tolerance was at 0.02 Da; the fixed modification was carbamidomethyl (C) and variable modification was oxidation (M); iTRAQ 8-plex was selected for quantification; only peptides with a false discovery rate (FDR) less than 1% were used for subsequent data analysis.

### 4.4. Bioinformatics Analysis

The identified proteins were annotated using the TAIR database (https://www.arabidopsis.org/, Fremont, CA, USA). The Kyoto Encyclopedia of Genes and Genomes (KEGG) pathway enrichment was performed using an online searching tool (http://www.omicsolution.org/wu-kong-beta-linux/passwd/KEGGEnrich/, Shanghai, China). The protein interaction analysis was performed using the String program (version 11.0, http://www.stringdb.org/, Hinxton, UK).

### 4.5. Plant Hormone Assay

Plant hormone contents were assayed using a published method [74]. 

### 4.6. Quantitative RT-PCR Analyses

The total RNA was extracted individually using 1mL of TRI reagent (Thermo Fisher Scientific, Waltham, MA, USA). For all samples, 2 μg of total RNA was converted to cDNA using M-MLV reverse transcriptase (Promega, Madison, WI, USA). Quantitative RT-PCR was performed with the Applied Biosystems 7500 RT-PCR system with SYBR Premix Ex *Taq* (Takara, Tokyo, Japan). The gene-specific primers (a single peak in qPCR melting curve products) used are listed in Table S4, and *ACTIN* was used as control. The relative quantification of RNA expression was calibrated using the formula 2^-ΔΔCt^ method. 

## 5. Conclusions

In this study, we investigated the effects of exogenous MeJA on Arabidopsis using the JA synthesis deficient mutant *opr3-1*. The differential defense against stress, photosynthesis, and development-related proteins were up-regulated in *opr3-1* after MeJA treatment, meanwhile, MeJA could also down regulate the expression of a large number of ribosomal proteins. Our study shows that, in the absent of endogenous JA, exogenous MeJA enhances Arabidopsis’ defense against stress, photosynthesis, and developmental processes, whilst also inhibiting protein synthesis process. For plant hormones, a trace level of JA could still be detected in *opr3-1*, indicating that the JA synthesis capability of the *opr3-1* mutant was significantly blocked but not completely inhibited, and we also found antagonism between JA and SA/ABA. The presented results provide a new framework and candidate protein list for further understanding the molecular mechanisms of exogenous JAs-regulated plant defense, photosynthesis, protein synthesis, and development process. 

## Supplementary Materials

Supplementary materials can be found at https://www.mdpi.com/1422-0067/21/2/571/s1. Figure S1. Workflow of the experiment. Figure S2. KEGG pathway enrichments of DRPs in MeJA-treated *opr3-1* compared with the *opr3-1* control group. Figure S3. Effects of MeJA treatment for 8 h on physiological parameters assays of two genotypes. (a)Water content; (b) Chlorophyll content; (c) H_2_O_2_ content; (d) POD content. Figure S4. Principle component analysis score plot of free amino acid contents of the two genotypes under MeJA treatment. Figure S5. Plant hormone contents in the two genotypes under MeJA treatment. (a) Jasmonic acid; (b) Abscisic acid; (c) Salicylic acid. Table S1. Details of differentially regulated proteins related to protein synthesis in *opr3-1*. Table S2. Details about the iTRAQ channels. Table S3. DRPs detected in Ws and *opr3-1* under MeJA treatment. Table S4. Primer sequences for gene expression analysis by RT-qPCR.

## Abbreviations

ABA	Abscisic acid
AOC	Allene oxide cyclase
AOS	Allene oxide synthase
DRPs	Differential regulated proteins
FDR	false discovery rate
GO	Gene ontology
H_2_O_2_	Hydrogen peroxide
IAA	Auxin
JA	Jasmonic acid
JA-Ile	Jasmonoyl-isoleucine
JAs	Jasmonates
JMT	Jasmonic acid carboxyl methyltransferase
KEGG	Kyoto Encyclopedia of Genes and Genomes
LOX	13-lipoxygenase
MeJA	Methyl jasmonate
OPR3	12-oxophytodienoic acid reductase 3
PCA	Principal component analysis
POD	Peroxidase
ROS	Reactive oxygen species
SA	Salicylic acid
STRING	Search Tool for the Retrieval of Interacting Genes
