# Supplementary material for: iTRAQ-Based Quantitative Proteomic Analysis of the Arabidopsis Mutant opr3-1 in Response to Exogenous MeJA
Source: Int J Mol Sci. 2020 Jan 16;21(2):571. doi: 10.3390/ijms21020571 (PMC7013738; doi:10.3390/ijms21020571)
Supplement: Supplementary file 1 [file ijms-21-00571-s001.zip › ijms-677100--supplementary/ijms-677100-Supplementary Materials.docx]

**
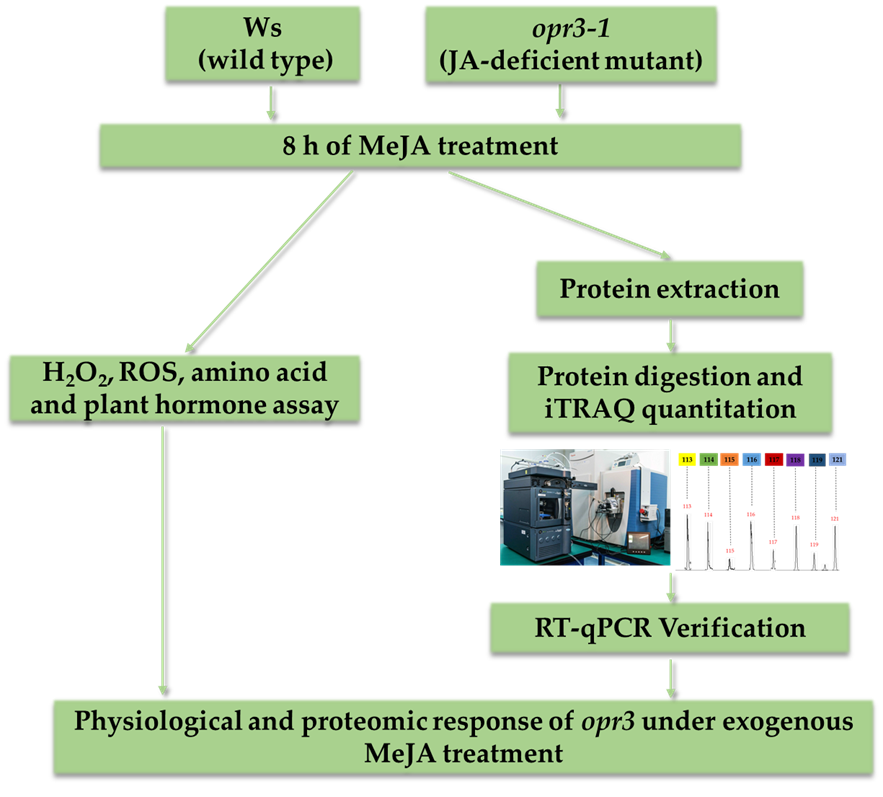
**

**Figure S1.** Workflow for the experiment.

**
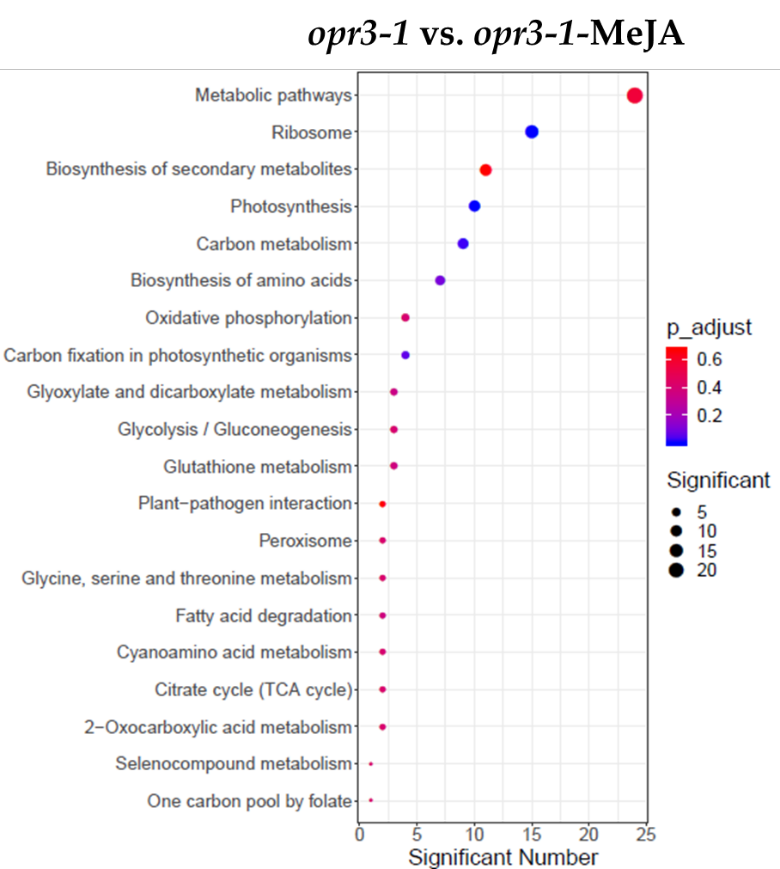
**

**Figure S2.** KEGG pathway enrichment of DRPs in MeJA-treated *opr3-1.*


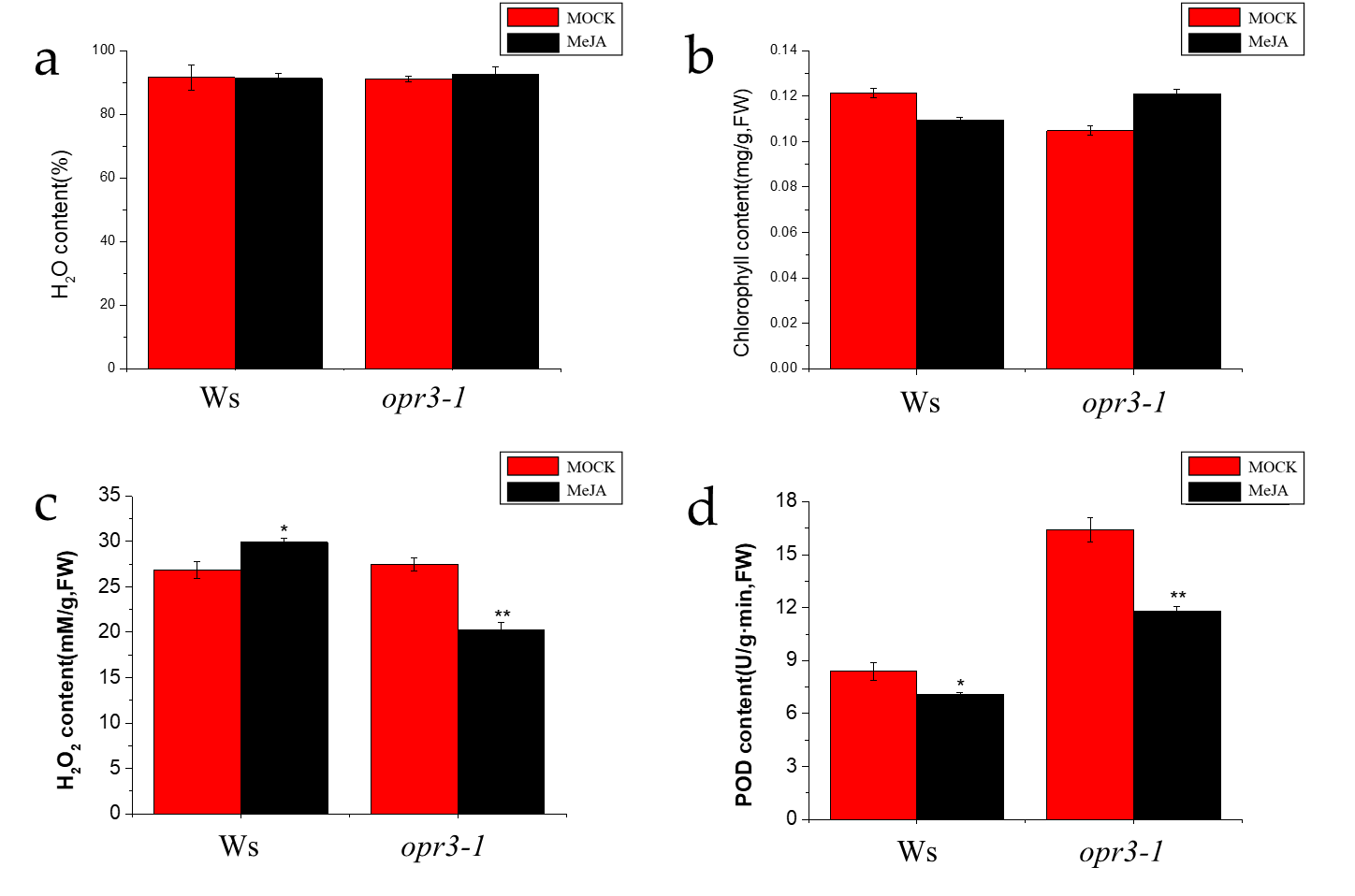


**Figure S3**. Effects of MeJA treatment on physiological parameters of the two genotypes. (**a**) Water content; (**b**) Chlorophyll content; (**c**) H_2_O_2_ content; (**d**) POD content.


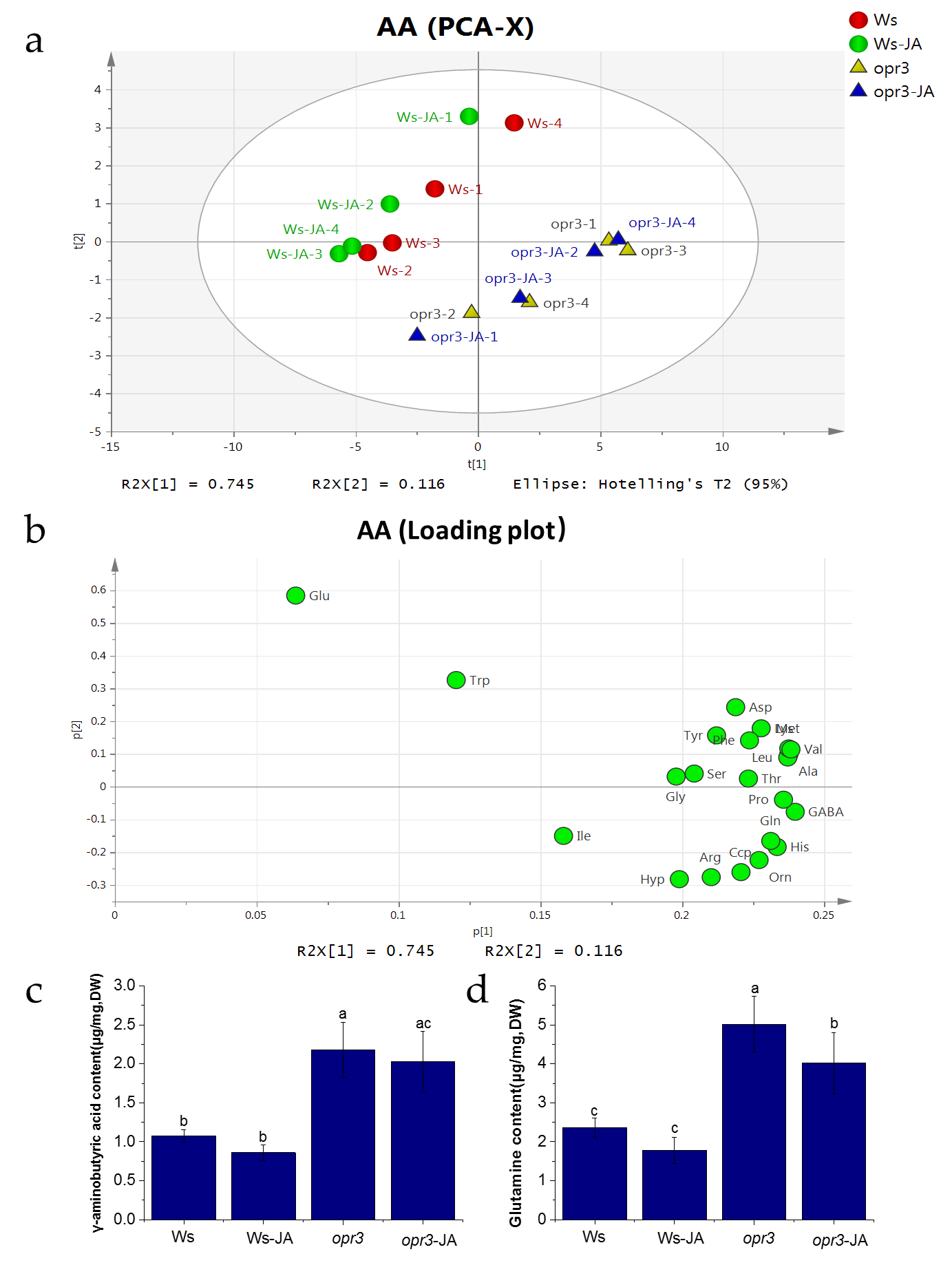


**Figure S4**. Principle component analysis score plot of free amino acid contents of the two genotypes under MeJA treatment.


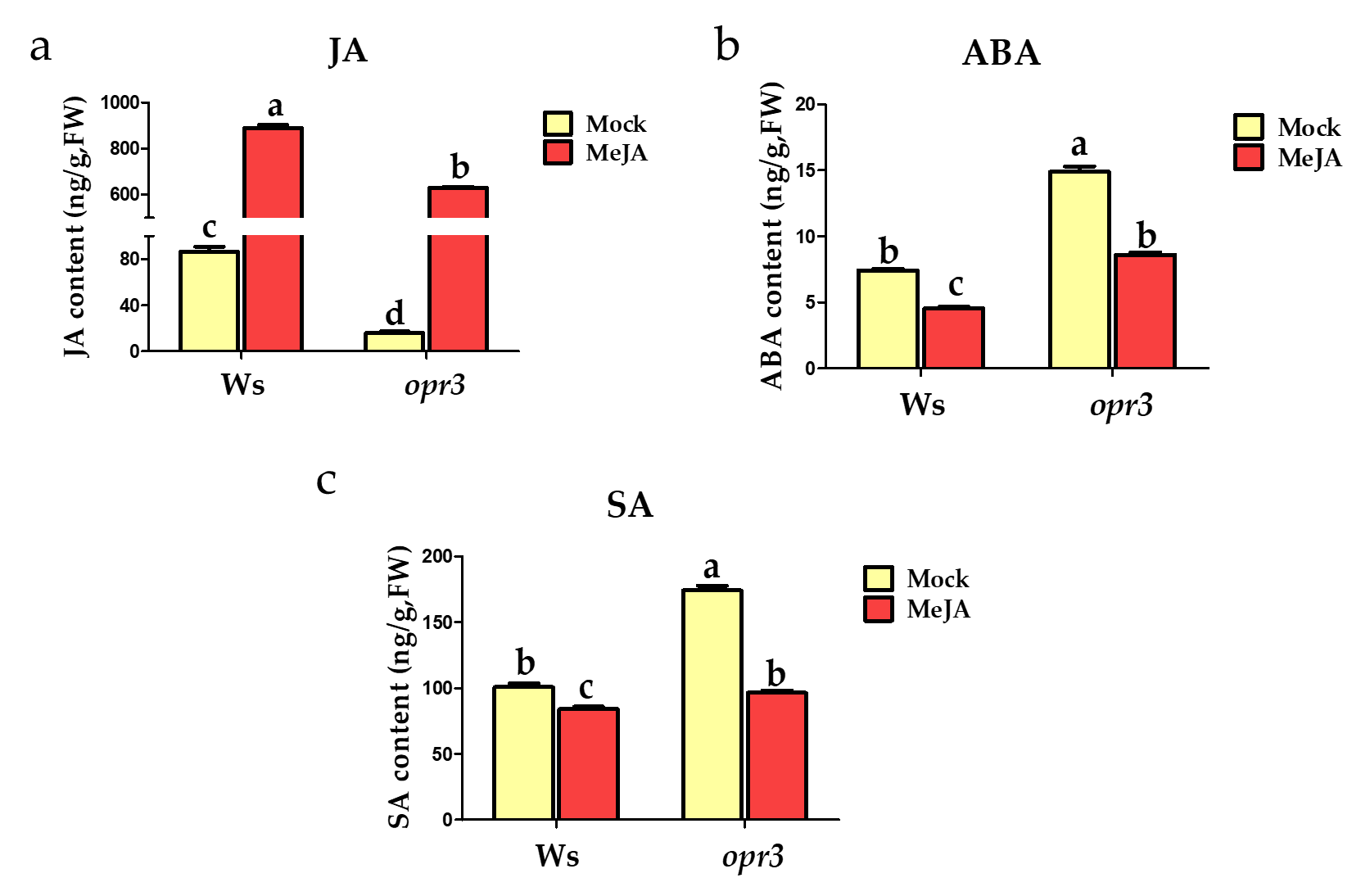


**Figure S5.** Plant hormone contents in the two genotypes under MeJA treatment. (**a**) Jasmonic acid; (**b**) Abscisic acid; (**c**) Salicylic acid.

**Table S1**. Details of DRPs related to ribosomal metabolism in *opr3-1.*

| **Accession** | **Protein name** | **Fold change**  **(*opr3-1-*JA/*opr3-1*)** | ***p*-Value** |
| --- | --- | --- | --- |
| **60S ribosomal proteins** | | | |
| AT4G15000.1 | 60S ribosomal protein L27-3 | 0.66 | 0.0024 |
| AT4G26230.1 | 60S ribosomal protein L31-2 | 0.64 | 0.0023 |
| AT3G05560.1 | 60S ribosomal protein L22-2 | 0.62 | 0.012 |
| AT4G27090.1 | **60S ribosomal protein L14-2** | 0.56 | 0.0052 |
| **50S ribosomal proteins** | | | |
| AT2G43030.1 | **50S ribosomal protein L3-1** | 0.66 | 0.0001 |
| **40S ribosomal proteins** | | | |
| AT2G41840.1 | **40S ribosomal protein S2-3** | 0.66 | 0.0001 |
| AT3G02080.1 | **40S ribosomal protein S19-1** | 0.65 | 0.013 |
| AT1G48830.1 | 40S ribosomal protein S7-1 | 0.61 | 0.0001 |
| AT3G02560.1 | 40S ribosomal protein S7-2 | 0.66 | 0.032 |
| AT5G02960.1 | **40S ribosomal protein S23-2** | 0.65 | 0.012 |
| **30S ribosomal proteins** | | | |
| ATCG00900.1 | **30S ribosomal protein S7** | 0.64 | 0.0001 |
| AT5G14320.1 | **30S ribosomal protein S13** | 0.66 | 0.006 |

**Table S2.** Details about the iTRAQ channels.

|  | Ws | Ws-MeJA | *opr3-1* | *opr3-1*-MeJA |
| --- | --- | --- | --- | --- |
| Replicate 1 | 113 | 114 | 115 | 116 |
| Replicate 2 | 117 | 118 | 119 | 121 |
| Replicate 3 | 121 | 119 | 118 | 114 |
